# Investigation of the Changes in the Power Distribution in Resting-State Brain Networks Associated with Pure Conduct Disorder

**DOI:** 10.1038/s41598-017-05863-3

**Published:** 2017-07-17

**Authors:** Jiang Zhang, Jiansong Zhou, Fengmei Lu, Liangyin Chen, Yunzhi Huang, Huafu Chen, Yutao Xiang, Gang Yang, Zhen Yuan

**Affiliations:** 1 Department of Medical Information Engineering, School of Electrical Engineering and Information, Sichuan University, Chengdu, 610065 China; 2 Mental Health Institute, Second Xiangya Hospital, Central South University, Hunan Province Technology Institute of Psychiatry, Key Laboratory of Psychiatry and Mental Health of Hunan Province, Changsha, 410011 China; 3 Bioimaging Core, Faculty of Health Sciences, University of Macau, Taipa, Macau SAR China; 4 School of Computer Science, Sichuan University, Chengdu, 610065 China; 5 School of Life Science and Technology, University of Electronic Science and Technology of China, Chengdu, 610054 China

## Abstract

Conduct disorder (CD) is a psychiatric disorder in children and adolescence. To investigate changes in the power distribution in brain networks between CD and typically developing (TD) groups, resting-state functional magnetic resonance imaging (rsfMRI) data of thirty-six subjects were first recorded, and then the data were preprocessed using DPARSF and SPM8. Meanwhile, the power of the blood oxygenation level-dependent (BOLD) signals of ninety brain regions was acquired using the integral of the Welch power spectral density (PSD). Additionally, the powers of the brain regions that reached significance (*p* < 0.05) were extracted using the bootstrap statistics, in which the standardized z-scores of the powers were used as a reference. The results of the analysis of the changes in power exhibited that there were significant power differences in some pairs of brain regions between the CD and TD groups, indicating a change in the power distribution. In addition, the results also suggest that the total power consumption of brain networks in CD patients is less than that observed in the TD group. Consequently, the study provided a paradigm for establishing quantifiable indicators via the power spectrum approach for the comparison and analysis of the BOLD signal power between CD patients and healthy controls.

## Introduction

Patients with conduct disorder (CD) may exhibit a repetitive and persistent pattern of aggressive and antisocial behaviors^1–3^. The symptoms of CD include but not limited to deception, theft, vandalism, violence, and serious violations of rules^1–4^. In addition, it is widely recognized that neural activity in the brain is accompanied by the changes in cerebral blood flow (CBF) and cerebral metabolic rate of oxygen (CMRO2), which can be measured by using the functional magnetic resonance imaging (fMRI) technique^5^. In the past years resting-state fMRI (rsfMRI) has attracted extensive attention regarding the measurement of spontaneous neuronal activity without any specific task, making it a useful and powerful technique for non-invasive mapping the hemodynamic responses in the brain during rest^6, 7^. More importantly, the blood oxygen level-dependent (BOLD) signals in fMRI recordings are due to the changes in CBF and CMRO2^5^. Consequently, the fluctuations of brain activity observed in the BOLD signals during the resting state play an essential role in exploring the neural mechanism of psychiatric and neurological disorders such as Alzheimer’s disease^8^ and epilepsy^9^. Interestingly, recent rsfMRI studies have been performed to explore the different brain activation patterns in CD^1–4, 10, 11^. However, most of work conducted focused on the investigation of the functional brain connectivity by examining the temporal correlation between the BOLD signals measured in different brain regions^12^.

In contrast to more commonly used functional connectivity analysis based on the correlation analysis between different brain regions, a power spectrum analysis method is proposed in this study, which allows us to investigate the oscillation power of brain regions. Power spectrum is a commonly used physical quantity that can quantitatively reflect the energy density changes of the object movement, which gives an intuitive sense of energy consumption changes in the dynamics. The adopted power spectrum method, such as the Welch spectrum method, has been validated to be able to improve the reliability of the analysis results of power spectral density (PSD)^13, 14^. In addition, it enables the neuroscientists to gain additional insight into the functional organization of the brain based on the generated power of BOLD signals within the whole brain regions. Based on the above reasons, we generated the hypothesis and concept for this study: 1) In resting state, whether or not the differences of BOLD signals in some brain regions are significant between CD and healthy subjects? 2) Can we use the improved power spectrum method to measure and quantitatively analyze the data from CD patients and healthy controls? 3) Can we identify the difference of brain region energy distributions between the two groups?

The purpose of the present study is to use the integrated power spectrum method to analyze the changes of the distribution of powers within different brain regions based on rsfMRI measurements. We will also examine whether the new paradigm can identify the difference of the powers of brain regions between young subjects with CD and young healthy controls. If succeeded, this model will provide us a tool towards an improved understanding of the neural mechanism of CD.

## Materials and Methods

### Subjects

Eighteen right-handed patients with pure CD (aged 15–17 years, males) were recruited from the Hunan province Youth Detention Center (YDC) in China. In addition, eighteen age-, gender-, and educational level-matched healthy subjects (typically developing (TD) group: aged 15–17 years, males) recruited from the community of Changsha, Hunan province and local schools participated in this study. By experimental design, none of the CD patients had current and lifetime comorbid psychiatric problems. More importantly, the K-SADS-PL (the Schedule for Affective Disorder and Schizophrenia for School-Age Children-Present and Lifetime)^15–17^, a semi-structured psychiatric interview based on DSM-IV criteria (the Diagnostic and Statistical Manual of Mental Disorders)^1^, was adopted in this study to exclude the additional psychiatric disorders by a professional interview psychiatrist. Consequently, all CD subjects met the K-SADS-PL criteria for CD and also the following criteria^18–21^: (1) satisfying the DSM-V criteria for CD; (2) no histories of neurological disorders; (3) no histories of other psychiatric disorders (e.g., attention deficit/hyperactivity disorder (ADHD), anxiety and depression disorders, affective disorders, obsessive-compulsive disorder, oppositional defiant disorder (ODD), mental retardation, alcohol- and drug-use disorder, and substance use disorder); (4) right handed; and (5) normal and corrected-to-normal vision. The subjects used for the present study were the same with the screened ones in the previous work^18^.

### Informed consent and ethical approval

All subjects, as well as their parents or caregivers, completed the written informed consent before the experimental tests. The protocol for all clinical trial was approved by the Biomedical Ethics Board of The Second Xiangya Hospital of Central South University and was carried out in accordance with the relevant guidelines, including any relevant details.

### Data acquisition

During the rsfMRI recordings, a foam padding with extendable padded head clamps was used to minimize the head motion, and the earplugs were used to minimize the effect of the scanning noise. All subjects were instructed to stay as still as possible, and to rest quietly with their eyes closed, and to relax without thinking of anything or falling asleep. The experimental tests were performed with a Siemens Allegra 3-T MR scanner at the Magnetic Resonance Center of Hunan Provincial People’s Hospital in Changsha, China. The echo planar imaging (EPI) settings were as follows: repetition time = 3.0 s; echo time = 30 ms; and flip angle = 90°. The contiguous axial slices aligned along the anterior commissure-posterior commissure line were acquired, and the imaging parameters were as follows: the number of slices = 36; field of view (FOV) = 256 mm × 256 mm; matrix size = 64 × 64; and slice thickness = 3 mm without a gap. For each subject, total 100 volume images were analyzed.

### Image pre-processing

The first 10 images for each subject were discarded to allow for steady-state longitudinal magnetization^22^. The remaining images were then preprocessed using DPARSF (http://restfmri.net/forum/DPARSF) and SPM8 (http://www.fil.ion.ucl.ac.uk/spm/) as follows: differences in image acquisition time between slices were corrected; the time-series of images were realigned to remove movement artifact; the images were normalized to a standard SPM8 EPI template which warps each individual subject into standard space with a resolution of 3 × 3 × 3 mm^3^ based on the Montreal Neurological Institute (MNI) template; and the images were smoothed with the full-width at half-maximum (FWHM) specified as 8 mm. Detrend and band-pass filtering (0.01~0.08 Hz) of the BOLD signals was performed to remove both low-frequency drift and high-frequency noise^23–25^. Additionally, the interferences were regressed out, which included the head motion parameters, white matter signal, cerebrospinal fluid signal and global mean signals.

### Data analysis

The functional brain images for each subject were mapped to the automated anatomical labeling (AAL) brain template, which were further separated into ninety anatomical brain regions within the cortex and subcortex (excluding cerebellum)^26^. The time series from all the voxels within each brain region were extracted and averaged, and then the mean BOLD signal for each brain region was generated. To access the power of each brain region, the Welch PSD^13, 14^ of the BOLD signal was required to be first calculated by using the PWELCH function in Matlab. The parameters of the function were set as follows: a 75-point Hamming window was used; the parameter on samples of overlap was omitted according to the window; and the function returned the one-sided PSD of the BOLD signal. According to the acquired PSD, the power of each brain region was generated by using the following equation,

1 $${P}_{i,j}={\int }_{f}{P}_{i,j}\,(f)df,$$

where *P*_*i*,*j*_ (*f*) and *P*_*i*,*j*_ are the Welch PSD and power of the *i*th brain region from the *j*th subject, respectively.

In addition, further processing was performed according to the power of the different brain regions:

1. Every brain region had a single power value calculated using eq. 1 and those regions with significant power were identified by the bootstrap statistics for each subject^27–31^.
2. Re-sampling was performed 5000 times to fit the normal distribution, and the mean and standard deviation of the distribution was calculated, in which the BOOTSTRP function in Matlab was utilized to implement this task. In particular, to obtain the power threshold corresponding to the statistical probability with *p-*value < 0.05, the inverse of the normal cumulative distribution with the corresponding mean and standard deviation needed to be calculated based on the following eqs 2 and 3. Here the inverse of the normal cumulative distribution was defined as:

2 $$x={F}^{-1}(x)=\{x:F(x)=P\}$$

where

3 $$P=F(x)=\frac{1}{\sqrt{2\pi }\sigma }{\int }_{-\infty }^{x}{e}^{\frac{-{(t-\mu )}^{2}}{2{\sigma }^{2}}}dt.$$

Based on the symmetry of a normal distribution, we set the probability *p* value as *2 P*. Meanwhile, *μ* and *σ* are the mean and standard deviation of the fitted normal distribution in bootstrap statistics, respectively. The NORMINV function in Matlab was used to calculate the inverse of the normal distribution, and the significance power threshold, corresponding to *p-*value < 0.05, was acquired using this function as well.

Consequently, for each subject we generated a very specific power threshold, and the power values of regions remained where they were greater than the corresponding threshold. In this way, the brain regions with the significant power were obtained for each subject (corresponding probabilities at *p-*value < 0.05). Then, the analysis returned the mean values of those significant powers for the eighteen subjects in the CD group and the brain regions with significant powers. The same operations were also performed for the TD group as well. The mean values of the significance powers from both groups were mapped to the AAL template, and the results were introduced into the BrainNet Viewer (http://www.nitrc.org/projects/bnv/)^32^ tool box to visualize the relationships between region structures and power network patterns with the significance level *p* < 0.05.

Further, the standardized indicators were used as a reference. *P*_*i*,*j*_ was standardized as follows:

4 $${Z}_{i,j}=({P}_{i,j}-mean({P}_{j}))/std({P}_{j}),$$

where *P*_*j*_ is the data vector including all powers of ninety brain regions from the *j*th subject and ${P}_{j}=({P}_{1,j},{P}_{2,j},{P}_{3,j},\cdots ,{P}_{90,j})$, the *mean*(*P*_*j*_) is the mean value of the data vector *P*_*j*_, and *std*(*P*_*j*_) is the standard deviation of *P*_*j*_. *Z*_*i*,*j*_ is the standardized z-scores of the *i*th brain region from the *j*th subject.

## Results and Discussion

**Figure 1:**
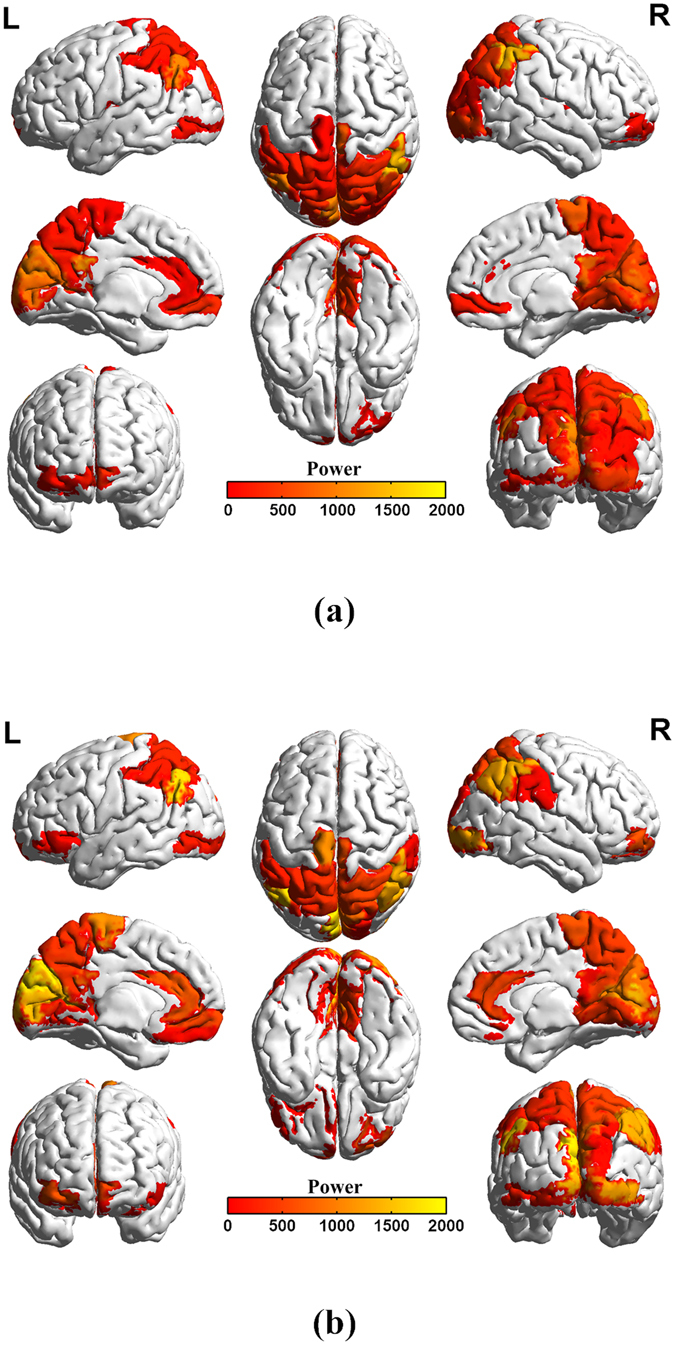
Network distribution of brain power with a nonparametric estimate *p* < 0.05: (**a**) CD; (**b**) TD. Panels (a,b) were generated by introducing the significant power values into the BrainNet Viewer (http://www.nitrc.org/projects/bnv/) tool box.

In this study, the bootstrap statistics was utilized to extract the significant power (*p* < 0.05) from ninety brain regions of each subject. Then, the mean values of the significant powers were generated for the eighteen subjects in the CD and TD group, respectively. The mean values and their distributions across different brain regions were compared between the CD and TD groups, in which Fig. 1 displayed the reconstructed three-dimensional (3D) network distribution by using the mean values of the significant powers. The relevant brain structural and functional information was provided in Table 1 for the power networks and associated brain regions in Fig. 1.

**Table 1:** Power of activated AAL regions of CD and TD groups in Fig. 1.

CD			TD		
Labels	Regions	Power	Labels	Regions	Power
10	Frontal_Mid_Orb_R	119.17	10	Frontal_Mid_Orb_R	657.27
25	Frontal_Mid_Orb_L	352.80	15	Frontal_Inf_Orb_L	88.44
26	Frontal_Mid_Orb_R	197.19	25	Frontal_Mid_Orb_L	566.23
31	Cingulum_Ant_L	79.73	27	Rectus_L	101.69
35	Cingulum_Post_L	1392.70	31	Cingulum_Ant_L	713.39
36	Cingulum_Post_R	842.46	32	Cingulum_Ant_R	547.83
43	Calcarine_L	880.23	35	Cingulum_Post_L	984.99
44	Calcarine_R	693.32	36	Cingulum_Post_R	92.00
45	Cuneus_L	1039.49	43	Calcarine_L	1465.98
46	Cuneus_R	548.32	44	Calcarine_R	1072.58
48	Lingual_R	427.67	45	Cuneus_L	1768.73
49	Occipital_Sup_L	356.00	46	Cuneus_R	860.37
50	Occipital_Sup_R	158.87	47	Lingual_L	196.98
52	Occipital_Mid_R	133.43	48	Lingual_R	420.04
53	Occipital_Inf_L	146.18	50	Occipital_Sup_R	107.02
54	Occipital_Inf_R	472.82	53	Occipital_Inf_L	233.73
59	Parietal_Sup_L	245.45	54	Occipital_Inf_R	1314.46
60	Parietal_Sup_R	341.73	59	Parietal_Sup_L	317.68
61	Parietal_Inf_L	182.36	60	Parietal_Sup_R	517.99
62	Parietal_Inf_R	1470.02	61	Parietal_Inf_L	227.09
65	Angular_L	1006.63	62	Parietal_Inf_R	1395.20
66	Angular_R	454.15	64	SupraMarginal_R	87.82
67	Precuneus_L	147.90	65	Angular_L	1681.29
68	Precuneus_R	232.89	66	Angular_R	1334.14
69	Paracentral_Lobule_L	69.21	67	Precuneus_L	394.65
70	Paracentral_Lobule_R	735.13	68	Precuneus_R	474.96
79	Heschl_L	97.49	69	Paracentral_Lobule_L	1116.60
80	Heschl_R	69.01	70	Paracentral_Lobule_R	658.14

The numbering sequence of labels is consistent with that of the ninety anatomical regions of interest in the AAL template.

To the best of our knowledge, power analysis was used for the first time to identify the differences of the brain activation patterns between the CD and TD groups. Interestingly, we discovered from Fig. 1 and Table 1 that both the CD and TD groups have twenty-eight brain regions with significant power, and most of the regions exhibited the similar distribution. However, this is not the case for other brain regions, in which brain activity with significant power was only identified in Frontal_Mid_Orb_R (Label 26), Occipital_Sup_L, Occipital_Mid_R, Heschl_L and Heschl_R for CD group, whereas only in the Frontal_Inf_Orb_L, Rectus_L, Cingulum_Ant_R, Lingual_L and SupraMarginal_R only existed for TD group. Importantly, the identified orbital gyri is involved in the cognitive processing of decision-making, and is thought to represent emotion and reward in decision making in individuals with CD relative to healthy controls^33–37^. In addition, previous work has revealed that the superior frontal gyrus including Frontal_Mid_Orb_R plays an essential role in higher levels of cognitive processing, such as working memory^11, 38^. In contrast, the superior/middle occipital gyrus including Occipital_Sup_L and Occipital_Mid_R are recognized to be related to the low-level perceptual systems and low-order cognitive processing^11, 39^. Meanwhile, our findings based on energy exhibited that there existed significant correlation between the brain regions and higher-order/low-order cognitive function processes in CD patients. In particular, it is widely recognized that the cingulum forms the white matter core of the cingulate gyrus and the anterior cingulate cortex is linked to emotion, especially apathy and depression. The changes of power can cause the behavioral change since the function of anterior cingulate cortex is correlated with emotions^40–42^. Further, the lingual gyrus plays an important role in processing vision, which is also associated with logical reasoning^43^ and encoding visual memories^44^. Interestingly, a recent study showed that brain activation in lingual gyrus and cuneus was negatively correlated with risk-taking in CD individuals^18, 45^. The decreased activity of right supramarginal gyrus can causes individuals to be more egocentric whereas overcoming emotional egocentricity bias is associated with increased activation in the regions^46^. In addition, the most significant activation regions based on the power (Fig. 1) were identified to be correlated with the activated components identified by the independent component analysis (ICA) from previous studies^11^. Interestingly, we also found that the largest power value in the brain regions from CD group was smaller than that of the TD group.

**Figure 2:**
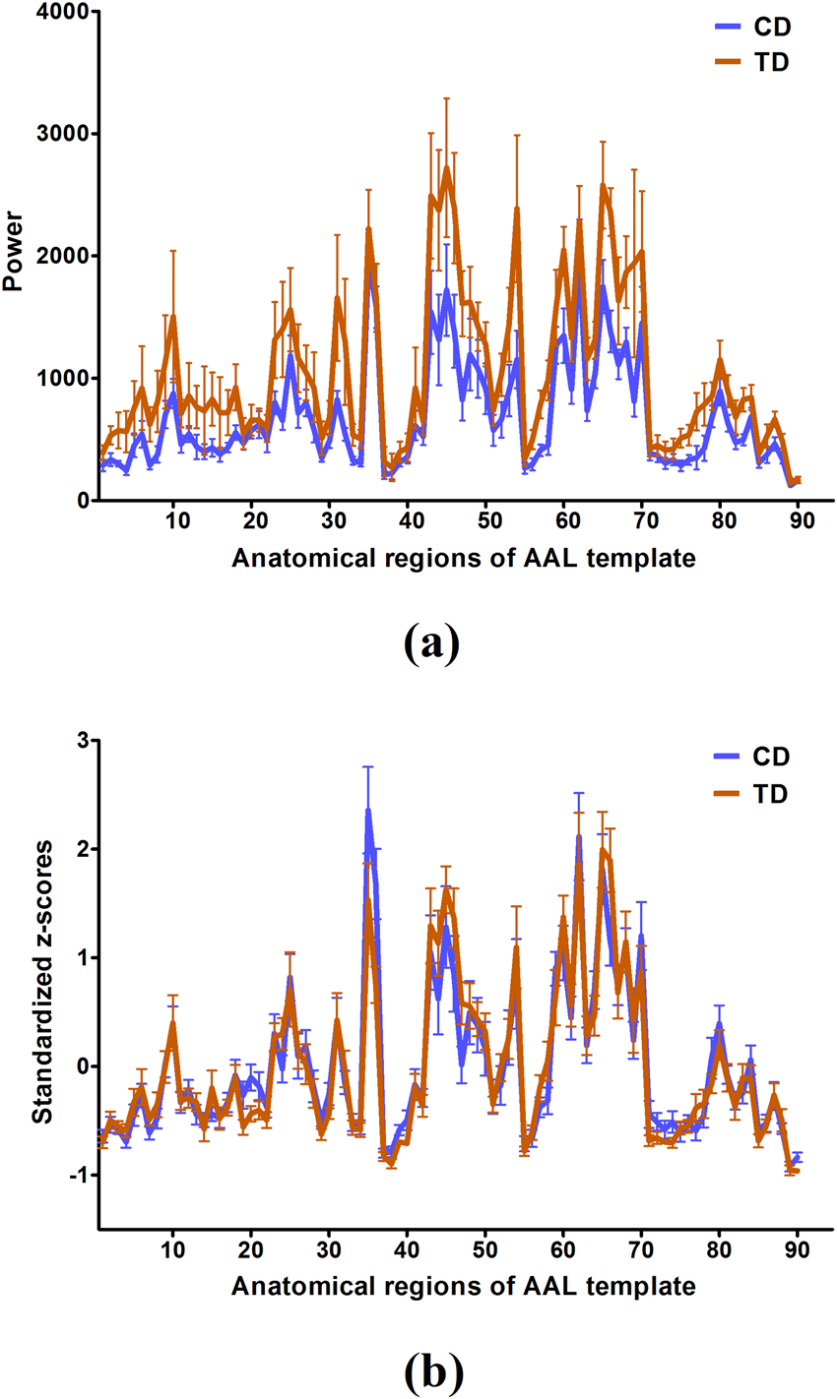
Power of brain regions and the standardized z-scores (mean ± SE): (**a**) Power curves (the unit for 10 log_10_ *Power* is decibels); (**b**) Standardized z-score curves. The blue color represents the CD group whereas the orange line represents the TD group. The horizontal axes denote the ninety anatomical regions of interest from the AAL template. The vertical axes in (**a**,**b**) denote the power of brain regions and standardized z-scores of power values, respectively.

More importantly, we also examined the power of the ninety brain regions for both CD and TD groups irrespective of whether the significant power was used or not. In Fig. 2(a), the blue color denoted the mean and standard error (SE) of the power of the brain regions from the eighteen subjects in the CD group, whereas the orange one represented those from the TD group. It was observed from Fig. 2 that the distribution of mean value of power from the CD group is similar to the TD group and the correlation coefficient between them is close to 0.93. In contrast, the sum of the power of the ninety brain regions from the CD group (61802.67) was smaller than that of the TD group (94131.96). In this figure, the numbers associated with the brain regions were used to simplify the figure, and the information of the labels of the ninety brain regions matching the AAL template was shown in Table 2. In addition, the standardized z-scores of the powers within the ninety brain regions were plotted in Fig. 2(b), in which we found that the distribution of mean value of the standardized power also exhibited the similarity between the CD and TD groups, and the correlation coefficient between them was 0.94.

**Table 2:** Labels of the ninety brain regions matching the AAL template.

Labels	Regions	Labels	Regions	Labels	Regions
1	Precentral_L	31	Cingulum_Ant_L	61	Parietal_Inf_L
2	Precentral_R	32	Cingulum_Ant_R	62	Parietal_Inf_R
3	Frontal_Sup_L	33	Cingulum_Mid_L	63	SupraMarginal_L
4	Frontal_Sup_R	34	Cingulum_Mid_R	64	SupraMarginal_R
5	Frontal_Sup_Orb_L	35	Cingulum_Post_L	65	Angular_L
6	Frontal_Sup_Orb_R	36	Cingulum_Post_R	66	Angular_R
7	Frontal_Mid_L	37	Hippocampus_L	67	Precuneus_L
8	Frontal_Mid_R	38	Hippocampus_R	68	Precuneus_R
9	Frontal_Mid_Orb_L	39	ParaHippocampal_L	69	Paracentral_Lobule_L
10	Frontal_Mid_Orb_R	40	ParaHippocampal_R	70	Paracentral_Lobule_R
11	Frontal_Inf_Oper_L	41	Amygdala_L	71	Caudate_L
12	Frontal_Inf_Oper_R	42	Amygdala_R	72	Caudate_R
13	Frontal_Inf_Tri_L	43	Calcarine_L	73	Putamen_L
14	Frontal_Inf_Tri_R	44	Calcarine_R	74	Putamen_R
15	Frontal_Inf_Orb_L	45	Cuneus_L	75	Pallidum_L
16	Frontal_Inf_Orb_R	46	Cuneus_R	76	Pallidum_R
17	Rolandic_Oper_L	47	Lingual_L	77	Thalamus_L
18	Rolandic_Oper_R	48	Lingual_R	78	Thalamus_R
19	Supp_Motor_Area_L	49	Occipital_Sup_L	79	Heschl_L
20	Supp_Motor_Area_R	50	Occipital_Sup_R	80	Heschl_R
21	Olfactory_L	51	Occipital_Mid_L	81	Temporal_Sup_L
22	Olfactory_R	52	Occipital_Mid_R	82	Temporal_Sup_R
23	Frontal_Sup_Medial_L	53	Occipital_Inf_L	83	Temporal_Pole_Sup_L
24	Frontal_Sup_Medial_R	54	Occipital_Inf_R	84	Temporal_Pole_Sup_R
25	Frontal_Mid_Orb_L	55	Fusiform_L	85	Temporal_Mid_L
26	Frontal_Mid_Orb_R	56	Fusiform_R	86	Temporal_Mid_R
27	Rectus_L	57	Postcentral_L	87	Temporal_Pole_Mid_L
28	Rectus_R	58	Postcentral_R	88	Temporal_Pole_Mid_R
29	Insula_L	59	Parietal_Sup_L	89	Temporal_Inf_L
30	Insula_R	60	Parietal_Sup_R	90	Temporal_Inf_R

**Figure 3:**
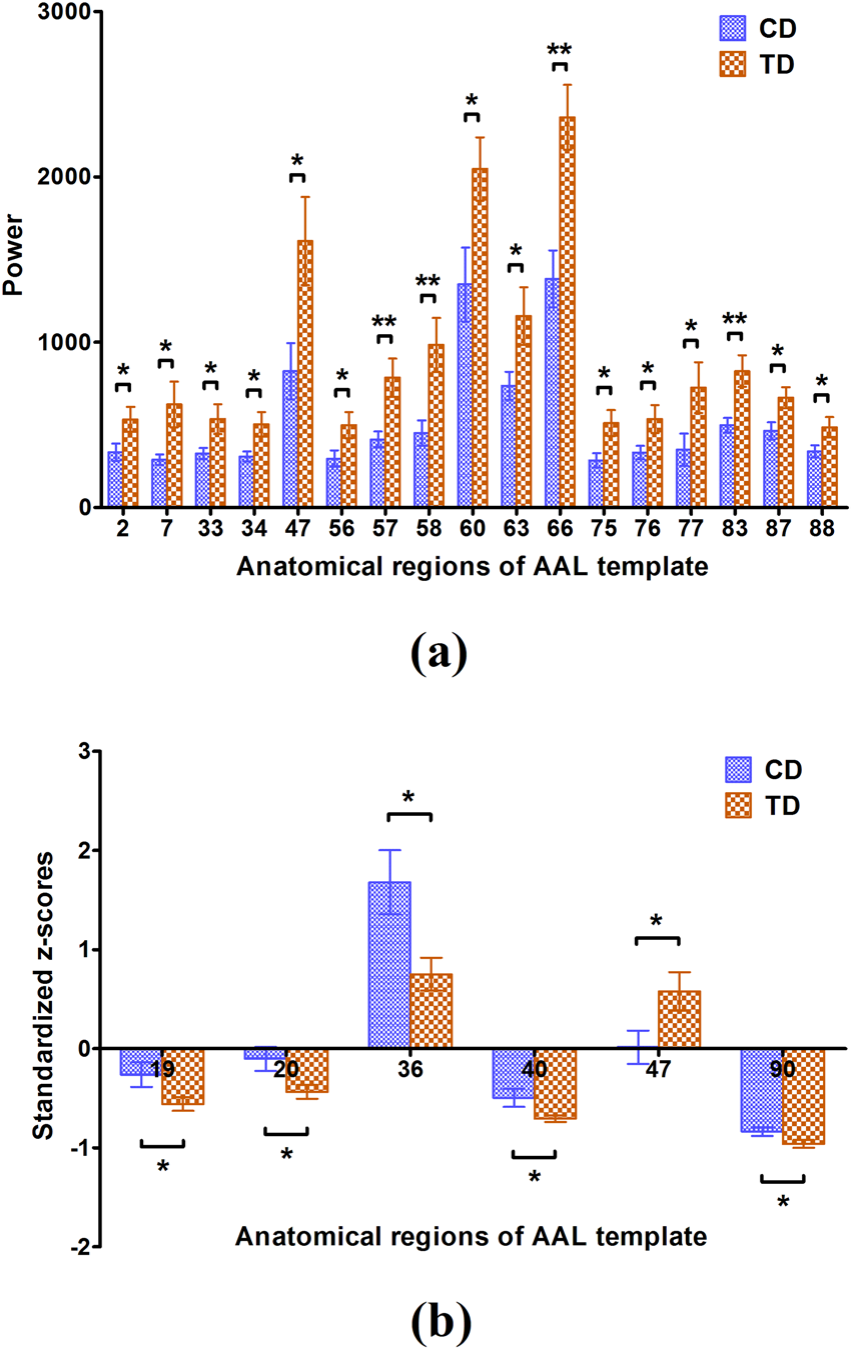
Brain regions with statistically significant differences between the CD and TD groups: (**a**) Power (mean ± SE); (**b**) Standardized z-scores (mean ± SE) of power. The blue color represents the CD group whereas the orange color represents the TD group; the horizontal axes denote the anatomical regions of interest in the AAL template. The vertical axes denote the power of brain regions in (**a**) and standardized z-scores of power values in (**b**), respectively. *p < 0.05 and **p < 0.01 (the *p* values are from two-sample t-tests between CD and TD).

Although there are great morphological similarities between the power curves of the CD and TD groups in Fig. 2, the correlation coefficients are less than 1. This suggests that power migration exists among the brain regions when comparing the CD with the TD group. Consequently, this study investigated the differences in the powers of brain regions between the CD and TD groups. Figure 3 showed the brain regions in which there was a statistically significant difference in power between the CD and TD groups. In particular, after powers were transformed into the standardized z-scores, there were six brain regions that exhibited significant differences between the CD and TD groups, which were displayed in Fig. 3(b). Importantly, the brain regions identified in Fig. 3(b) are different from those revealed in Fig. 3(a).

**Figure 4:**
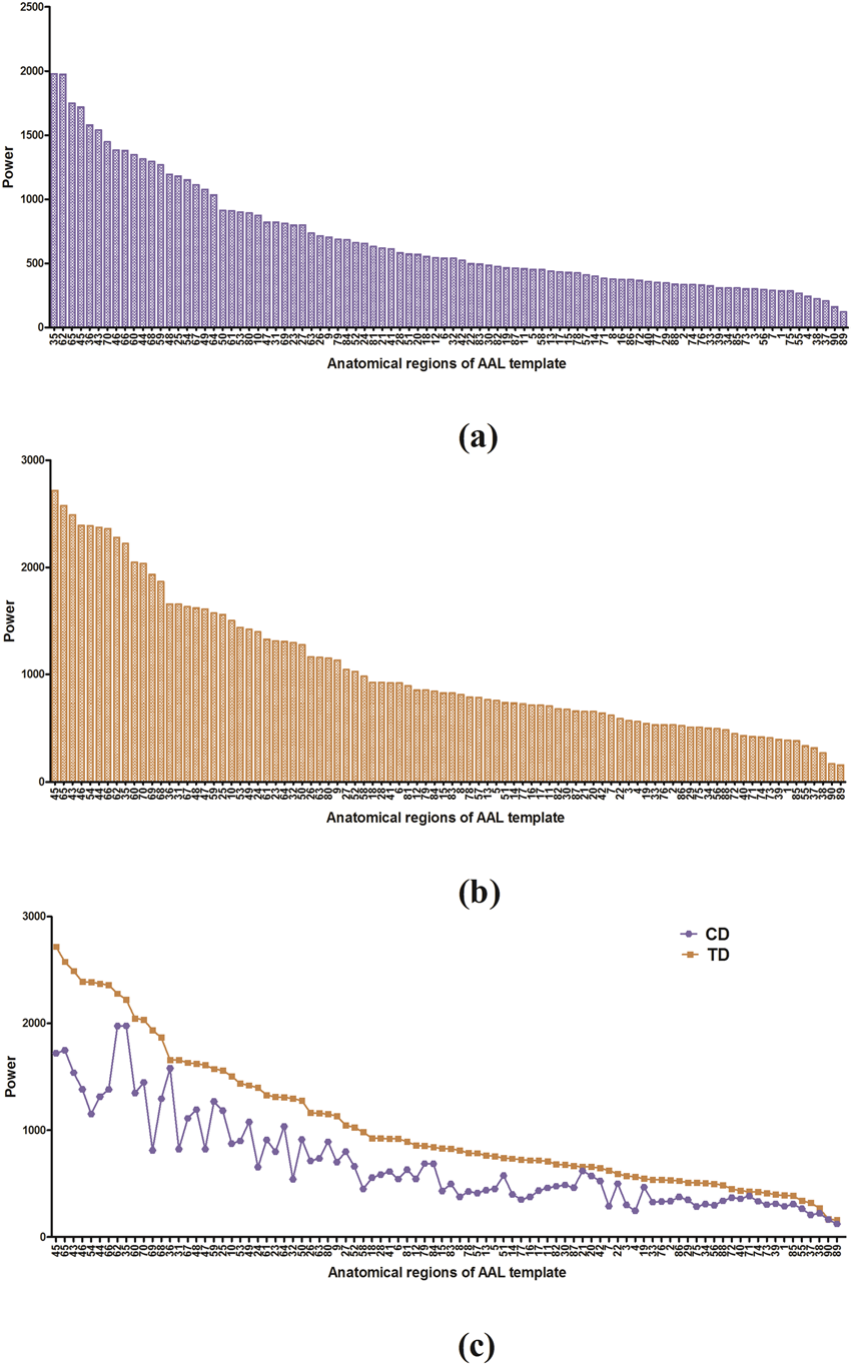
Power distribution and comparison. CD group in (**a**) and TD group in (**b**) express the power distribution of brain regions in descending order of power.; (**c**) expresses the comparison between CD and TD groups with the order of brain regions in panel (b).

To survey the change in the distribution of power within the ninety brain regions in the CD and TD groups, we also calculated the mean power intensity for each of the ninety brain regions for the subjects from the CD and TD group, respectively. Then, the brain regions were sorted by the power intensity in descending order, which were shown in Fig. 4. We discovered from Fig. 4 that the powers of brain regions in the CD group were smaller than that in the TD group, and the order of the brain regions changed from the TD group in (b) to the CD group in (a).

The power analysis of the BOLD signals was performed to examine the energy consumption difference between the CD and TD groups. Interestingly, the new power spectrum approach involves the analysis of the power of the BOLD signal across the whole frequency bands rather than several frequencies, which can acquire more unique features of patients in the CD group. The power of fMRI BOLD signals directly reflects the degree of energy consumption within a region, which is able to quantify the energy consumption during a certain period of time. In this study, we explored the mean power of each brain region using the integrals of power spectral densities (PSDs) of the BOLD signals, in which PSDs were calculated by using the Welch spectrum method^13, 14^. The Welch method allows for the selection of a variety of window functions and improving the calculation of the PSD (PWELCH is a Matlab function which calculates the PSD using the Welch’s method). To balance the variance and resolution by reducing the sensitivity of the PSD to noise in the BOLD signals, the function was set to use the default segment parameters in Matlab, and then the BOLD signal was automatically segmented into eight sections of equal length, each with 50% overlap based on a Hamming window.

We think it is reliable to adopt the power spectrum to detect the abnormal brain functions. Firstly, the power spectrum of fMRI time series has been used to analyze brain functional activation in previous studies^47–51^, which can quantitatively determine the power changes of BOLD signals. As such, the previous work exhibited that the power spectrum analysis approaches are effective and reproducible in the analysis of BOLD signals. In particular, the Welch power spectrum used in this study is able to cover a wide variety of window functions, which can improve the quality of spectrum to an even better degree, and is recognized to be an accepted classical power spectrum estimation approach. And the brain power analysis is quite different from the correlation analysis since the power and correlation coefficient are two distinct measurements. The power analysis results in this study directly reflects the degree of energy consumption within the brain regions while the correlation coefficient from two time series is indicative of the functional connectivity between two nodes. In addition, the power distribution analysis uses the integral of the Welch power spectral density (PSD) to measure the power consumption of brain networks, and to compare the BOLD signal power between patients and healthy subjects. The method is also different from reported regional homogeneity (ReHo) analysis^52–54^. ReHo is used to measure the temporal synchrony of regional blood oxygen level-dependent (BOLD) signals as well as similarities in spontaneous neural activity^52–54^. It assumes that within a functional cluster, the hemodynamic characteristics of each voxel would be similar, or synchronous with that of each other, and such similarity could be changed or modulated by different conditions^52, 54^. Technically, ReHo uses the Kendall’s coefficient concordance (KCC) to measure the similarity of the time series of a given voxel to those of its nearest neighbors in a voxel-wise way^52^.

As it is very hard to recruit the CD subjects, only 18 patients were identified to participate in this study. Meanwhile, 18 heathy controls were also invited to take part in this work to balance the size of two samples. The small sample size may affect the statistical power for determining neural marks of CD although our findings indeed exhibit the significant difference in power between the two groups based on the small size sample. Although most brain regions with significant power were identified to be the same between the CD and TD groups, as displayed in Fig. 1 and Table 1, a few of the ninety brain regions analyzed did exhibit the difference between them. This indicates that a significant change in the distribution of power occurred between the two groups in a few of the ninety brain regions analyzed. The results shown in Fig. 1 and Table 1 also indicated that the significant powers for most of the brain regions in the CD group were lower than those of the TD group. Although these brain regions showed significant power during the resting state, most of the regions with significant power in the CD group were calmer than those in the TD group. Since the brain regions can interrelate and influence each other^55, 56^, we analyzed the relation of the powers in the ninety brain regions. In Fig. 2(a), by comparing the means of powers of the eighteen subjects from the CD group with that of the TD group, we discovered that the powers of the ninety brain regions in the TD group were larger than that in the CD group during the resting state, which implies that the brains of the TD group displayed a higher amount of activity. Further, the standardized z-score chart in Fig. 2(b) showed that the distributions of the two mean curves are highly similar. Thus, we further tested the statistically significant differences between the powers of brain-region pairs between the CD and TD groups. This study found that the powers of some brain-region pairs were significantly different between the CD and TD groups during resting state as shown in Fig. 3. The mean values of powers from the TD group were larger than that of the CD group during the resting state (shown in Fig. 3(a)), and statistically significant differences between standardized z-scores of the powers in the brain regions is also indicative of a change in the distribution of power between the TD and CD groups (shown in Fig. 3(b)). Moreover, we also sorted the brain regions of the TD and CD groups according to the strength of the power (shown in Fig. 4). The comparison of Fig. 4(a–c) reflect the change in power observed between the CD and TD groups. Based on these observed changes in the power in the brain regions of the CD group compared to the TD group, we can infer that functional activities related to these brain regions are observed in CD patients and the adjustment of these changes in power may provide a therapeutic strategy for the improvement and rehabilitation of CD. Clinically, the measure of power represents a specific level of brain activity, which can be used to identify the brain regions associated with different disorders or brain cognition functions, and to reflect the energy consumption within brain regions. These power indicators or neural marks can provide us additional clinical supplementary reference information for disease diagnosis and treatments.

## Conclusions

Power spectrum estimation is one of the classic methods to analyze neural signals. In this study, power spectrum method was used to extract and analyze the power of functional brain regions in the CD and TD groups based on rsfMRI recordings. We discovered that there were significant differences in the power observed in brain regions in the CD and TD groups, indicative of a change in the distribution of power between the two groups, and the total power of the regions analyzed in the CD group was less than that of the TD group. The study provided a new paradigm for establishing quantifiable indicators via the power spectrum approach for the comparison and analysis of the BOLD signal power between patients and healthy subjects.
